# Heme oxygenase 2 genetic variants alter hormonal and metabolic traits in polycystic ovary syndrome

**DOI:** 10.1530/EC-23-0463

**Published:** 2024-02-12

**Authors:** Xinyuan Zhang, Suiyan Li, Hongwei Liu, Huai Bai, Qingqing Liu, Chunyi Yang, Ping Fan

**Affiliations:** 1 Laboratory of Genetic Disease and Perinatal Medicine, Key Laboratory of Birth Defects and Related Diseases of Women and Children, Ministry of Education, West China Second University Hospital, Sichuan University, Chengdu, Sichuan, China; 2 School of Life Science and Engineering, Southwest Jiaotong University, Chengdu, Sichuan, China; 3 Department of Obstetrics and Gynecology, West China Second University Hospital, Sichuan University, Chengdu, Sichuan, China

**Keywords:** polycystic ovary syndrome, heme oxygenase 2, genetic polymorphism, metabolism, oxidative stress

## Abstract

Oxidative stress and metabolic disorders are involved in the pathogenesis of polycystic ovary syndrome (PCOS). Heme oxygenase 2 (HMOX2) plays a critical role in preserving heme metabolism as well as in modulating glycolipid metabolism, oxidative stress, and inflammation. This study examined the correlation between *HMOX2* G554A (rs1051308) and A-42G (rs2270363) genetic variants with the risk of PCOS and assessed the effects of these genotypes on clinical, hormonal, metabolic, and oxidative stress indices using a case–control design that included 1014 patients with PCOS and 806 control participants. We found that the allelic and genotypic frequencies of the HMOX2 *G554A* and A-42G polymorphisms were comparable between the PCOS and control groups in Chinese women (*P* > 0.05). Nevertheless, it was discovered that patients with the AA or AG genotype of A-42G polymorphism had notably elevated levels of estradiol (E_2_), follicle-stimulating hormone (FSH), luteinizing hormone (LH), LH/FSH ratio, high-density lipoprotein cholesterol (HDL-C), total cholesterol (TC), low-density lipoprotein cholesterol (LDL-C), apolipoprotein (apo)B, and/or apoB/apoA1 ratio than those with the GG genotypes (*P* < 0.05). Patients with the GG or AG genotype of G554A polymorphism had elevated serum levels of LH, FSH, E_2_, LH/FSH ratio, TC, HDL-C, LDL-C, apoB, and/or apoB/apoA1 ratio and lower 2-h glucose concentration compared with those with the AA genotype (*P* < 0.05). Our findings indicate a potential association between the genetic variants and endocrine abnormalities in the reproductive system and metabolic irregularities in glycolipid levels in patients, thus suggesting their potential role in the pathogenesis of PCOS.

## Introduction

Polycystic ovary syndrome (PCOS) is a common endocrine disorder that affects approximately 10–13% of women in their adolescent, reproductive, and postmenopausal years (1). PCOS is characterized by clinical and/or biochemical hyperandrogenism, oligo-ovulation or anovulation, which result in irregular menstrual cycles, and/or polycystic ovarian morphology. It is often associated with various cardiometabolic and psychological abnormalities, including overweight or obesity, insulin resistance, heightened oxidative stress, low-grade chronic inflammation, dyslipidemia, increased risks of complications during pregnancy, type 2 diabetes, cardiocerebrovascular disease, anxiety and depression, obstructive sleep apnea, and endometrial cancer (1, 2, 3, 4). The etiology of PCOS remains unclear; however, growing evidence suggests that it may have a diverse and intricate etiology that includes interactions between a variety of predisposing genes and environmental factors (5, 6, 7, 8).

Heme oxygenase (HMOX) is the rate-limiting enzyme that catalyzes the degradation of iron protoporphyrin heme to produce ferrous ions (Fe^2+^), carbon monoxide (CO), and biliverdin (BV) (9, 10). BV is subsequently metabolized to bilirubin (BR) by BV reductase and expelled from the cells (9, 11). The products of HMOX enzymatic activity, BV and its metabolite BR, are potent inhibitors of oxidative stress-mediated tissue damage (9, 11). CO can mediate vasodilation and possesses antiapoptotic, antiproliferative, antithrombotic, and anti-inflammatory properties, while free Fe^2+^ is a pro-oxidant (9, 10). HMOX mainly includes two isomers, inducible HMOX1 and constitutive HMOX2, which demonstrate similar structure and catalytic properties; they play crucial roles in regulating the level and bioavailability of heme (11). In view of the effects of heme and its catabolites on cell physiology, HMOX activity may affect cellular metabolism and pathophysiological processes (9, 11, 12, 13).

Human* HMOX2* is located on chromosome 16p13.3 (14). HMOX2 is expressed in almost all tissues and cells, particularly brain, testes, and endothelial cells (10, 15). HMOX2 is barely regulated at the transcriptional and translational levels; however, it still plays a vital role in heme homeostasis, antioxidation, anti-inflammation, and metabolic regulation (10, 11, 13, 15). Single-nucleotide polymorphisms (SNPs) in *HMOX2* are likely related to variations in HMOX2 enzymatic activity and function (16, 17). It has been reported that *HMOX2* rs1051308 (*G554A*) SNP is associated with the risk of essential tremors (18) and multiple sclerosis (19), while *HMOX2* rs2270363 (*A-42G*) SNP is related to the risk of schizophrenia (20) and age-related macular degeneration (21). Additionally, both SNPs are also relevant to Parkinson’s disease (17, 22).

Oxidative stress and metabolic disorders play a considerable role in the pathophysiology and progress of PCOS (4, 7, 23, 24). However, the association of *HMOX2* genetic variants with PCOS remains unknown. Therefore, we investigated the association between *HMOX2* G554A and A-42G genetic variants and susceptibility to PCOS and assessed the effects of the genotypes on the clinical, metabolic, and oxidative stress parameters in a well-characterized Chinese population with a substantial sample size (1014 cases and 806 controls).

## Materials and methods

### Study participants

This case–control study included 1014 patients diagnosed with PCOS and 806 healthy control women. Between 2006 and 2022, individuals aged 17–40 years were enrolled at the Outpatient Clinic of Reproductive Endocrinology at the West China Second University Hospital. All participant provided written informed consent, and the study followed the guidelines of the Declaration of Helsinki. This study was approved by the Institutional Review Board of West China Second University Hospital, Sichuan University (2014-014 for Ping Fan).

Individuals were diagnosed with PCOS according to the revised 2003 Rotterdam ESHRE/ASRM consensus criteria (25), which included the following: (i) irregular menstrual cycles, either oligo-ovulation or anovulation (OA), were defined as menstrual cycles shorter than 21 or longer than 35 days or as less than eight cycles per year (1); (ii) biochemical and/or clinical hyperandrogenism (HA) was defined as serum total testosterone (TT) ≥2.60 nmol/L and/or free androgen index (FAI) ≥9.5 greater than 95th percentile in menstruating women with regular cycles, hirsutism with a modified Ferriman–Gallwey (F-G) score ≥6, and/or moderate-severe acne (26, 27, 28); and (iii) polycystic ovaries (PCOs), which were defined as at least 12 follicles of diameter of 2–9 mm in each ovary and/or ovarian volume >10 mL on ultrasonography. PCOS was diagnosed if a patient met at least two of the three criteria after ruling out other causes, such as androgen-secreting tumors, Cushing syndrome, and congenital adrenal hyperplasia (1). Additionally, HA and irregular menstrual cycles were prerequisites for individuals aged <20 years (1). Participants in the control group had regular menstrual cycles that ranged from 21 to 35 days without clinical signs of hirsutism or acne and had normal serum levels of androgens as well as normal ovarian morphology on ultrasonography.

Participants with other diseases, such as infections, liver or kidney diseases, cardiovascular conditions, autoimmune/inflammatory disorders, thyroid disorders, hypogonadotropic hypogonadism, hyperprolactinemia, malignancies, premature ovarian insufficiency, or endometriosis were excluded.

To determine the genetic links between HMOX2 genetic variants and the risk of PCOS, 1014 patients with PCOS and 806 healthy controls were included. To analyze oxidative stress, hormonal, and metabolic indices, participants were excluded if they had any of the following factors: (i) hormonal therapy (oral contraceptives) or medications known to affect glycolipid metabolism (e.g. metformin) within 3 months before the study; (ii) pregnancy or luteal menstrual phase, which can also affect glycolipid metabolism and oxidative stress (29, 30); (iii) smoking; and/or (iv) fasting glucose (Glu) ≥ 7.0 mmol/L and/or 2-h Glu ≥ 11.1 mmol/L in the control group. Finally, 661 patients and 516 controls were included in this study.

Clinical variables, such as body mass index (BMI, kg/m^2^), waist circumference (WC), waist-to-hip ratio, systolic and diastolic blood pressure (SBP and DBP, respectively), and the extent of hirsutism and acne were assessed as described previously (27, 28). The volume of the ovaries was calculated using the following ellipsoid formula (31): 0.523 × width (cm) × length (cm) × thickness (cm).

After fasting for 8–12 h, blood samples were obtained, promptly placed on ice, and centrifuged at 1500 ***g*** for 15 min at 4°C within 2 h. The blood cells were preserved at 4°C, and serum and plasma were frozen at −80°C for subsequent analysis. After fasting blood sampling, a 75 g oral glucose tolerance test was instantly conducted.

### DNA extraction and genotyping

Genomic DNA was extracted from the blood cells (32). *HMOX2* G554A and A-42G SNPs were identified using polymerase chain reaction (PCR) amplification and restriction analysis. Primer-BLAST was utilized to design the PCR amplification primers for G554A SNP (forward primer: 5′-GATGCTGCTTCCGGTAGTCC-3′ and reverse primer: 5′-GCTTCTGAGGGACTCTCCTATG-3′) and A-42G SNP (forward primer: 5′-GAAGCACGCCTACTTACCCC-3′ and reverse primer: 5′-AGATGACACGCCCCCTTGTAG-3′). PCRs were performed as follows: initial denaturation at 95°C for 3 min followed by 37 cycles of 45 s at 95°C, 45 s at 58°C, and 45 s at 72°C for G554A, and 36 cycles of 30 s at 95°C, 30 s at 60°C, and 30 s at 72°C for A-42G, and ending with a single extension step of 7 min at 72°C. Five microliters PCR products of G554A SNP (375 bp) or 3.5 μL A-42G SNP (125 bp) were digested using 4 U of Hhal or 2 U of NlaIII (New England Biolabs, Inc.) in a final 10 µL reaction volume for 2–16 h at 37 °C, respectively. Digestion resulted in 262 bp and 113 bp fragments representing the 554G allele, an undigested 375 bp fragment representing the 554A allele, 72 bp and 53 bp fragments representing the -42G allele, and an undigested 125 bp fragment representing the -42A allele. The digested fragments were examined using electrophoresis on agarose gel (2.5 or 3.5%) and Genecolor fluorescent dye. For quality assessments, more than 25% of the DNA samples were randomly regenotyped by a different experimenter, and the results of both experiments were identical.

### Evaluation of parameters related to oxidative stress, hormones, and metabolism

Serum total oxidant status (TOS), malondialdehyde (MDA), total antioxidant capacity (T-AOC), oxidative stress index (OSI), TT, estradiol (E_2_), luteinizing hormone (LH), follicle-stimulating hormone (FSH), sex hormone-binding globulin (SHBG), free androgen index (FAI), HDL-cholesterol (HDL-C), LDL-cholesterol (LDL-C), total cholesterol (TC), triglycerides (TG), apolipoprotein (apo) A1, and apoB levels, plasma Glu and insulin (Ins) concentrations, and the homeostatic model assessment of insulin resistance (HOMA-IR) were determined or evaluated as previously described (4, 23, 27, 28).

Plasma glutathione (GSH) levels were determined using the microplate fluorescence method based on the cyclic reaction of 2,3-naphthalenedicarboxaldehyde (NDA) with the glutamyl amino groups of GSH and cysteine sulfhydryl, resulting in a cyclized product with high fluorescence; GSH was used as the standard. The assay was performed according to the method by White *et al.* (33) with few modifications. Briefly, 80 μL of phosphate-buffered saline (PBS, pH7.2) and 40 μL of triscarboxyethylphosphine (TCEP) solution (1 mM, prepared immediately before use) were combined with 20 μL of plasma, and the resulting mixture was placed on ice for 15 min. Subsequently, 100 μL of 5% 5-sulfosalicylic acid dehydrate (SSA) was added to the mixture, incubated on ice for 15 min, and then centrifuged at 10,000 ***g*** for 2 min at 4 °C. Thirty-five microliters of the supernatant and 25 μL of GSH calibrators (0, 0.25, 0.5, 1.0, 1.5, 2.0, and 2.5 μmol/L in PBS containing 2.5% SSA) were added to flat-bottomed, black, 96-well polystyrene microtiter plates (Thermo Scientific Nunc, Roskilde, Denmark). Next, we added 100 μL of 0.1 M *n*-ethlymorpholine (NEM)/0.02 M KOH solution to each well and 10 μL of TCEP solution to each GSH calibrator well. The blend was incubated for 15 min at room temperature (20–25 °C). Subsequently, 50 μL of 0.5 M NaOH solution and 10 μL of 10 mM NDA solution (prepared immediately before use) were added to each well, gently mixed, and left to incubate at room temperature for 30 min without light. Next, the plate was then positioned on a Varioskan Flash Multimode Microplate Spectrophotometer (Thermo Scientific), gently mixed, and the fluorescence intensity was measured at an excitation wavelength of 472 nm and emission wavelength of 528 nm, with a wave width of 5 nm. The concentration of plasma GSH, which was expressed in μmol/L, was obtained using computerized data reduction of the fluorescence values for the calibrators *vs.* the final concentrations of the reaction using a four-parameter logistic model. Sample duplicates were plated, and a mixed serum sample was added to each plate for quality control.

The coefficients of variation for all determinations, both within and between assays, were less than 5% and 10%, respectively.

### Statistical analysis

Data are presented as mean ± s.d. for symmetric variables or median (25th–75th percentile value) for asymmetric variables. The differences in variables between the PCOS and control groups or genotype subgroups were compared using the independent sample t-test or analysis of variance (ANOVA) for the normally distributed variables before or after logarithmic transformation, and the Mann–Whitney *U* test for asymmetric variables. Analysis of covariance (ANCOVA) was used to assess disparities in clinical and biochemical indices (logarithmic transformation was performed for asymmetric variables before analysis) between the two groups or subgroups after adjusting for age and BMI diversity. Chi-squared (*χ*^2^) analysis was used to analyze the deviations of genotypic distribution from the Hardy–Weinberg parameters and the genotypic and allelic frequencies between patients and controls. A *P*-value less than 0.05 was deemed to be statistically significant. The SPSS® 21.0 (IBM SPSS Statistic, IBM Corporation) was used for data analysis.

Using the online THEsis software (http://analysis.bio-x.cn/myAnalysis.php), we estimated the matrix of haplotype frequencies and linkage disequilibrium between various genetic polymorphic loci based on the D’ parameter.

## Results

### Clinical and biochemical characteristics

Age was lower and BMI was higher in the PCOS group than in the control group (Table 1); therefore, subsequent comparisons of all other parameters between the two groups or subgroups were corrected for these differences.

**Table 1:** Clinical, hormonal, metabolic, and oxidative stress parameters in patients with PCOS and control women.

	Controls (*n* = 806)	PCOS (*n* = 1014)	*P*	*P^a^*
**Clinical parameters**				
Age (years)	28.25 ± 4.11	25.06 ± 4.18	<0.001	
BMI (kg/m^2^)	21.14 ± 2.84	22.89 ± 4.04	<0.001	
WC (cm)	73.72 ± 8.11	79.09 ± 11.03	<0.001	<0.001
Waist-to-hip ratio	0.82 ± 0.06	0.84 ± 0.07	<0.001	<0.001
F-G score	0.25 ± 0.74	1.72 ± 2.04	<0.001	<0.001
Acne grade score	0.14 ± 0.34	0.65 ± 0.89	<0.001	<0.001
SBP (mm Hg)	112.59 ± 11.23	114.25 ± 10.60	0.002	0.528
DBP (mm Hg)	73.57 ± 8.76	75.50 ± 8.71	<0.001	0.010
Ovarian volume (mL)	7.43 ± 2.75	9.93 ± 4.06	<0.001	<0.001
**Hormonal levels^b^**				
E_2_ (pmol/L)	287.44 (177.68, 528.99)	214.75 (164.05, 309.84)	0.245	0.895
TT (nmol/L)	1.47 ± 0.52	2.33 ± 0.78	<0.001	<0.001
SHBG (nmol/L)	50.90 (35.10, 67.10)	30.10 (19.40, 44.70)	<0.001	<0.001
FAI	2.68 (1.85, 4.18)	7.97 (4.87, 11.62)	<0.001	<0.001
LH (IU/L)	4.80 (3.30, 7.20)	10.80 (6.20, 16.00)	<0.001	<0.001
FSH (IU/L)	6.56 ± 2.62	6.01 ± 2.12	<0.001	0.027
LH/FSH	0.97 (0.65, 1.46)	1.96 (1.24, 2.82)	<0.001	<0.001
**Metabolic profile^b^**				
Fasting Ins (pmol/L)	55.56 (39.59, 78.97)	85.91 (55.56, 128.83)	<0.001	<0.001
2 h-Ins (pmol/L)	303.91 (197.83, 472.71)	568.45 (355.76, 974.46)	<0.001	<0.001
Fasting Glu (mmol/L)	5.23 ± 0.47	5.34 ± 0.81	0.002	0.768
2 h-Glu (mmol/L)	5.96 ± 1.27	7.10 ± 2.35	<0.001	<0.001
HOMA-IR	1.95 (1.41, 2.75)	2.90 (1.86, 4.47)	<0.001	<0.001
TG (mmol/L)	0.88 (0.68, 1.18)	1.09 (0.80, 1.67)	<0.001	<0.001
TC (mmol/L)	4.24 ± 0.72	4.42 ± 0.81	<0.001	<0.001
HDL-C (mmol/L)	1.52 ± 0.33	1.38 ± 0.34	<0.001	0.003
LDL-C (mmol/L)	2.35 ± 0.63	2.56 ± 0.76	<0.001	<0.001
ApoA1 (g/L)	1.46 ± 0.21	1.43 ± 0.21	0.003	0.959
ApoB (g/L)	0.75 ± 0.17	0.83 ± 0.20	<0.001	<0.001
ApoB/apoA1	0.52 ± 0.14	0.59 ± 0.18	<0.001	<0.001
**Oxidative stress parameters^b^**				
TOS (nmol H_2_O_2_ Equiv./mL)	10.22 (7.96, 13.91)	12.17 (9.07, 17.14)	<0.001	<0.001
T-AOC (U/mL/min)	14.50 ± 2.67	15.79 ± 3.06	<0.001	<0.001
OSI	0.70 (0.51, 0.98)	0.75 (0.57, 1.04)	<0.001	<0.001
MDA (nmol/mL)	3.70 ± 1.10	4.35 ± 1.29	<0.001	<0.001
GSH (nmol/mL)	1.11 ± 0.25	1.18 ± 0.25	<0.001	0.010
TOS/GSH	8.98 (7.05, 12.13)	10.35 (7.39, 14.01)	<0.001	<0.001

Values are presented as mean ± s.d. or median (25th–75th percentile value).

^a^Comparisons of the parameters were corrected for differences in age and BMI between the two groups; ^b^Controls (*n* = 516), PCOS (*n* = 661).

apoA1, apolipoprotein A1; apoB, apolipoprotein B; BMI, body mass index; DBP, diastolic blood pressure; E_2_, estradiol; FAI, free androgen index; F-G, Ferriman–Gallwey; FSH, follicle-stimulating hormone; Glu, glucose; GSH, glutathione; HDL-C, high-density lipoprotein cholesterol; HOMA-IR, index homeostatic model assessment of insulin resistance; Ins, insulin; LDL-C, low-density lipoprotein cholesterol; LH, luteinizing hormone; MDA, malondialdehyde; OSI, oxidative stress index; SBP, systolic blood pressure; SHBG, sex hormone-binding globulin; T-AOC, total antioxidant capacity; TC, total cholesterol; TG, triglycerides; TOS, total oxidant status; TT, total testosterone; WC, waist circumference.

As shown in Table 1, the PCOS group had significantly higher WC, waist-to-hip ratio, acne grade score, F-G score, DBP, average ovarian volume, TT, FAI, LH, LH/FSH ratio, fasting Ins, HOMA-IR, 2 h-Ins and Glu, TC, LDL-C, TG, apoB, apoB/apoA1 ratio, T-AOC, TOS, OSI, GSH, MDA, and TOS/GSH ratio but lower SHBG, FSH, and HDL-C levels compared to the control group.

### Frequencies of *HMOX2* genotype, allele, and haplotype

In both the PCOS and control groups, the genotypic distributions of* HMOX2* A-42G and G554A SNPs were consistent with Hardy–Weinberg equilibrium (all *P* > 0.05).

The different genetic models of* HMOX2* A-42G and G554A SNPs are summarized in Table 2. Genotype, dominant, recessive, and allele models for A-42G and G554A polymorphisms of *HMOX2* did not reveal any significant differences between the PCOS and control groups.

**Table 2:** Association of *HMOX2* A-42G and G554A polymorphisms with the risk of PCOS using different genetic models.

	Controls (*n* = 806)	PCOS (*n* = 1014)	*χ*^2^	*P*
**A-42G**				
Genotype				
GG	330 (40.9%)	418 (41.2%)		
AG	373 (46.3%)	476 (46.9%)		
AA	103 (12.8%)	120 (11.8%)	0.378	0.828
Recessive				
AA	103 (12.8%)	120 (11.8%)		
GG + AG	703 (87.2%)	894 (88.2%)	0.373	0.541
Dominant				
GG	330 (40.9%)	418 (41.2%)		
AA + AG	476 (59.1%)	596 (58.8%)	0.015	0.905
Allele				
G	1033 (64.1%)	1312 (64.7%)		
A	579 (35.9%)	716 (35.3%)	0.147	0.701
**G554A**				
Genotype				
AA	341 (42.3%)	430 (42.4%)		
AG	363 (45.0%)	465 (45.9%)		
GG	102 (12.7%)	119 (11.7%)	0.380	0.827
Recessive				
GG	102 (12.7%)	119 (11.7%)		
AA + AG	704 (87.3%)	895 (88.3%)	0.356	0.551
Dominant				
AA	341 (42.3%)	430 (42.4%)		
GG + AG	465 (57.7%)	584 (57.6%)	0.002	0.966
Allele				
A	1045 (64.8%)	1325 (65.4%)		
G	567 (35.2%)	703 (34.6%)	0.130	0.718

Data are presented as numbers (%). Genetic models (genotype, recessive, dominant, and allele models).

The haplotype distributions of *HMOX2* A-42G and G554A SNPs are shown in Table 3. Among the four haplotypes, two common haplotypes (frequency >0.03, Ht2 and Ht3) accounted for 97.6% and 98.1% of the observed haplotypes in patients with PCOS and controls, respectively. The haplotype distribution was similar between the PCOS and control groups (*P* = 0.688). A relatively strong linkage disequilibrium was found between A-42G and G554A SNPs (D’ = 0.968, *r* ^2^ = 0.909).

**Table 3:** Proportions of haplotypes of *HMOX2* A-42G and G554A SNPs in the PCOS and control groups.

Haplotype	Locus		No. (% frequency)		OR	95% CI	*χ*^2^	*P*
	A-42G	G554A	Control	PCOS				
Ht1	A	A	21 (0.013)^a^	30 (0.015)^a^	1.141	0.652–1.997	0.214	0.643
Ht2	A	G	558 (0.346)	686 (0.338)	0.965	0.841–1.108	0.252	0.616
Ht3	G	A	1024 (0.635)	1295 (0.638)	1.014	0.8485–1.162	0.041	0.389
Ht4	G	G	9 (0.006)^a^	17 (0.009)^a^	1.509	0.675–3.376	1.018	0.313

Haplotypes data are presented as number (%) of patients or controls. Linkage disequilibrium coefficients (D’ = 0.968, *r*^2^ = 0.909) between the two SNPs in *HMOX2*. Global *χ*^2^ = 0.161, Fisher’s *P* = 0.688.

^a^Because four cells had less than 3% haplotype frequency, Fisher’s exact test was used.

CI, confidence interval; OR, odds ratio.

### Effects of *HMOX2* A-42G and G554A SNPs on clinical and biochemical variables

Table 4 reveals that patients with the AA genotype of A-42G polymorphism exhibited notably elevated levels of E_2_, LH, FSH, and HDL-C levels than those with the GG or AG genotype (*P* < 0.05) as well as higher E_2_, LH/FSH ratio, TC, LDL-C, and apoB levels than those with the GG genotype (*P* < 0.05). Patients with the AG genotype had higher LDL-C, apoB, and apoB/apoA1 ratio compared with those with the GG genotype (*P* < 0.05). Controls with the AA genotype of A-42G SNP had significantly higher apoB/apoA1 ratio but lower F-G score in comparison with those with the GG or AG genotype (*P* < 0.05), had lower FAI than those with the GG genotype (*P* = 0.037), and lower apoA1 levels than those with the AG genotype (*P* = 0.028). Controls with the AG genotype also had higher TG levels and OSI in comparison with those with the GG genotype (*P* < 0.05).

**Table 4:** Clinical, hormonal, metabolic, and oxidative stress parameters of the* HMOX2* A-42G genotype in the PCOS and control women.

	Controls			PCOS		
	GG (*n* = 330)	AG (*n* = 373)	AA (*n* = 103)	GG (*n* = 418)	AG (*n* = 476)	AA (*n* = 120)
** Clinical parameters**						
Age (years)	28.26 ± 4.17	28.26 ± 4.15	28.17 ± 3.79	25.16 ± 4.71	25.01 ± 4.31	24.93 ± 3.92
BMI (kg/m^2^)	20.98 ± 2.72	21.25 ± 2.91	21.24 ± 2.99	23.03 ± 3.97	22.76 ± 4.06	22.92 ± 4.22
WC (cm)	73.74 ± 8.09	73.67 ± 8.12	73.85 ± 8.19	79.42 ± 10.95	78.87 ± 10.87	78.77 ± 11.95
Waist-to-hip ratio	0.81 ± 0.06	0.81 ± 0.06	0.82 ± 0.06	0.85 ± 0.07	0.85 ± 0.07	0.84 ± 0.07
F-G score	0.27 ± 0.74	0.29 ± 0.82	0.07 ± 0.29^a,b^	1.73 ± 2.10	1.72 ± 1.99	1.72 ± 2.04
Acne grade score	0.13 ± 0.34	0.15 ± 0.36	0.10 ± 0.30	0.63 ± 0.87	0.67 ± 0.91	0.60 ± 0.87
SBP (mm Hg)	112.72 ± 11.43	112.63 ± 11.05	112.04 ± 11.31	114.68 ± 10.73	113.98 ± 10.42	113.86 ± 10.87
DBP (mm Hg)	73.97 ± 8.58	73.37 ± 8.95	72.97 ± 8.68	75.26 ± 8.66	75.79 ± 8.79	75.19 ± 8.57
Ovarian volume (mL)	7.07 ± 2.42	7.69 ± 3.11	7.67 ± 2.49	10.09 ± 3.96	9.88 ± 4.34	9.55 ± 3.32
**Hormonal levels^e^**						
E_2_ (pmol/L)	216.22 (161.16, 346.54)	222.46 (151.25, 310.29)	211.08 (148.13, 386.56)	204.29 (158.22, 263.21)	207.41 (161.16, 281.57)	237.15 (173.27, 355.35)^c^
TT (nmol/L)	1.45 ± 0.53	1.49 ± 0.49	1.43 ± 0.60	2.35 ± 0.83	2.29 ± 0.72	2.41 ± 0.82
SHBG (nmol/L)	48.80 (34.55, 64.95)	52.20 (34.80, 68.35)	53.80 (39.85, 72.95)	29.90 (18.50, 44.70)	28.65 (18.80, 42.60)	31.10 (20.50, 42.80)
FAI	2.68 (1.85, 4.07)	2.68 (1.90, 4.19)	2.55 (1.44, 3.51)^a^	8.14 (5.10, 11.61)	8.05 (5.06, 11.63)	8.02 (5.05, 12.83)
LH (IU/L)	5.20 (3.50, 8.25)	5.40 (4.00, 8.30)	5.60 (3.90, 7.85)	11.50 (7.20, 16.50)	12.05 (7.80, 16.30)	12.80 (9.70, 19.40)^c,d^
FSH (IU/L)	6.61 ± 2.99	6.50 ± 2.31	6.63 ± 2.42	5.97 ± 1.74	5.90 ± 1.89	6.54 ± 3.48^c,d^
LH/FSH	0.83 (0.59, 1.28)	0.92 (0.65, 1.40)	0.92 (0.64, 1.23)	2.00 (1.32, 2.70)	2.01 (1.42, 2.96)	2.17 (1.53, 3.10)^c^
**Metabolic profile^e^**						
Fasting Ins (pmol/L)	53.48 (40.28, 73.97)	58.34 (39.59, 79.17)	52.64 (41.19, 77.34)	90.15 (57.65, 133.24)	86.12 (55.77, 125.91)	86.53 (59.73, 128.76)
2 h-Ins (pmol/L)	288.77 (202.69, 414.20)	300.09 (182.21, 483.13)	292.04 (169.53, 378.92)	565.67 (353.61, 1098.08)	558.66 (323.78, 969.17)	516.02 (331.59, 1103.70)
Fasting Glu (mmol/L)	5.21 ± 0.53	5.23 ± 0.45	5.26 ± 0.37	5.33 ± 0.78	5.34 ± 0.87	5.37 ± 0.66
2 h-Glu (mmol/L)	6.01 ± 1.21	5.93 ± 1.34	5.92 ± 1.20	7.35 ± 2.55	6.89 ± 2.17	7.03 ± 2.18
HOMA-IR	1.82 (1.41, 2.54)	1.94 (1.36, 2.82)	1.85 (1.41, 2.71)	2.99 (1.91, 4.61)	2.91 (1.89, 4.36)	2.88 (2.00, 4.67)
TG (mmol/L)	0.81 (0.65, 1.08)	0.92 (0.73, 1.22)^a^	0.91 (0.60, 1.36)	1.09 (0.81, 1.73)	1.11 (0.80, 1.68)	0.99 (0.74, 1.45)
TC (mmol/L)	4.21 ± 0.82	4.24 ± 0.63	4.36 ± 0.73	4.37 ± 0.78	4.42 ± 0.84	4.56 ± 0.76^c^
HDL-C (mmol/L)	1.52 ± 0.32	1.52 ± 0.34	1.51 ± 0.32	1.38 ± 0.36	1.35 ± 0.33	1.46 ± 0.33^c,d^
LDL-C (mmol/L)	2.31 ± 0.68	2.34 ± 0.57	2.47 ± 0.66	2.49 ± 0.73	2.59 ± 0.79^c^	2.66 ± 0.70^c^
ApoA1 (g/L)	1.46 ± 0.22	1.48 ± 0.21	1.42 ± 0.19^b^	1.42 ± 0.21	1.43 ± 0.22	1.43 ± 0.21
ApoB (g/L)	0.74 ± 0.19	0.75 ± 0.15	0.78 ± 0.17	0.81 ± 0.19	0.84 ± 0.21^c^	0.85 ± 0.20^c^
ApoB/apoA1	0.52 ± 0.15	0.52 ± 0.13	0.56 ± 0.15^a,b^	0.58 ± 0.17	0.60 ± 0.19^c^	0.61 ± 0.18
**Oxidative stress parameters^e^**						
TOS (nmol H_2_O_2_ Equiv./mL)	9.45 (7.62, 12.97)	10.21 (7.76, 13.97)	11.40 (8.63, 15.04)	12.26 (9.02, 16.77)	11.68 (9.05, 16.82)	10.97 (8.50, 17.36)
T-AOC (U/mL/min)	14.43 ± 2.53	14.52 ± 2.86	14.66 ± 2.32	15.66 ± 3.16	15.90 ± 3.02	15.83 ± 2.85
OSI	0.66 (0.50, 0.86)	0.71 (0.51, 1.02)^a^	0.81 (0.56, 1.04)	0.77 (0.57, 1.03)	0.74 (0.60, 1.03)	0.77 (0.53, 1.11)
MDA (nmol/mL)	3.64 ± 1.14	3.74 ± 1.01	3.72 ± 1.25	4.37 ± 1.27	4.30 ± 1.29	4.48 ± 1.38
GSH (nmol/mL)	1.11 ± 0.24	1.11 ± 0.25	1.12 ± 0.25	1.19 ± 0.26	1.18 ± 0.24	1.18 ± 0.24
TOS/GSH	8.74 (6.71, 11.22)	8.97 (7.02, 12.67)	9.71 (7.38, 12.47)	10.31 (7.41, 13.81)	10.41 (7.60, 14.29)	9.32 (6.82, 14.28)

Values are presented as mean ± s.d. or median (25th–75th percentile value). Comparisons of all parameters between two subgroups were corrected for differences in age and BMI except the parameters of age and BMI.

^a^*P* < 0.05, compared with the GG genotype subgroup in the control group; ^b^*P* < 0.05, compared with the AG genotype subgroup in the control group; ^c^*P* < 0.05, compared with the GG genotype subgroup in the PCOS group; ^d^*P* < 0.05, compared with the AG genotype subgroup in the PCOS group; ^e^Controls (GG = 213, AG = 238, AA = 65); PCOS (GG = 272, AG = 301, AA = 88).

apoA1, apolipoprotein A1; apoB, apolipoprotein B; BMI, body mass index; DBP, diastolic blood pressure; E_2_, estradiol; FAI, free androgen index; F-G, Ferriman–Gallwey; FSH, follicle-stimulating hormone; Glu, glucose; GSH, glutathione; HDL-C, high-density lipoprotein cholesterol; HOMA-IR, index homeostatic model assessment of insulin resistance; Ins, insulin; LDL-C, low-density lipoprotein cholesterol; LH, luteinizing hormone; MDA, malondialdehyde; OSI, oxidative stress index; SBP, systolic blood pressure; SHBG, sex hormone-binding globulin; T-AOC, total antioxidant capacity; TC, total cholesterol; TG, triglycerides; TOS, total oxidant status; TT, total testosterone; WC, waist circumference.

Regarding G554A SNP (Table 5), patients with the GG genotype demonstrated significantly elevated FSH and HDL-C levels compared with those with the AA or AG genotype (*P* < 0.05) and increased E_2_, LH, LH/FSH ratio, TC, and LDL-C levels relative to those with the AA genotype (*P* < 0.05). Patients with the AG genotype also had higher apoB levels and apoB/apoA1 ratio (*P* < 0.05) but lower 2 h-Glu concentrations (*P* = 0.042) than those with the AA genotype. Controls with the GG genotype had a significantly higher apoB/apoA1 ratio than those with the AA or AG genotype (*P* < 0.05). Controls with the AG genotype also had a higher LH/FSH ratio, TG levels, and OSI (*P* < 0.05), but lower WC (*P* = 0.039) than those with the AA genotype.

**Table 5:** Clinical, hormonal, metabolic, and oxidative stress parameters of the *HMOX2 G554A* genotype in the PCOS and control women.

	Controls			PCOS		
	AA (*n* = 341)	AG (*n* = 363)	GG (*n* = 102)	AA (*n* = 430)	AG (*n* = 465)	GG (*n* = 119)
** Clinical parameters**						
Age (years)	28.32 ± 4.15	28.18 ± 4.17	28.26 ± 3.80	25.09 ± 4.13	25.03 ± 4.25	25.11 ± 4.15
BMI (kg/m^2^)	21.02 ± 2.73	21.21 ± 2.88	21.29 ± 3.10	23.06 ± 3.99	22.65 ± 3.99	23.20 ± 4.36
WC (cm)	73.84 ± 8.14	73.52 ± 8.03^a^	74.06 ± 8.34	79.49 ± 10.96	78.63 ± 10.75	79.43 ± 12.33
Waist-to-hip ratio	0.81 ± 0.06	0.81 ± 0.06	0.82 ± 0.06	0.85 ± 0.07	0.85 ± 0.07	0.85 ± 0.07
F-G score	0.28 ± 0.75	0.27 ± 0.79	0.11± 0.49	1.72 ± 2.10	1.73 ± 1.99	1.70 ± 2.04
Acne grade score	0.14 ± 0.35	0.14 ± 0.35	0.10 ± 0.30	0.65 ± 0.88	0.66 ± 0.90	0.59 ± 0.88
SBP (mm Hg)	112.59 ± 11.31	112.66 ± 11.25	112.34 ± 10.99	114.86 ± 10.88	113.75 ± 10.16	114.07 ± 11.26
DBP (mm Hg)	73.82 ± 8.57	73.45 ± 9.03	73.13 ± 8.50	75.35 ± 8.89	75.73 ± 8.57	75.13 ± 8.61
Ovarian volume (mL)	7.07 ± 2.39	7.71 ± 3.16	7.67 ± 2.49	10.08 ± 3.93	9.90 ± 4.36	9.49 ± 3.36
**Hormonal levels^e^**						
E_2_ (pmol/L)	216.22 (162.00, 346.54)	222.83 (151.07, 308.63)	211.08 (148.13, 386.56)	202.46 (154.54, 255.69)	212.59 (162.08, 288.91)	236.78 (173.27, 350.95)^c^
TT (nmol/L)	1.44 ± 0.52	1.51 ± 0.50	1.43 ± 0.59	2.33 ± 0.82	2.33 ± 0.75	2.36 ± 0.75
SHBG (nmol/L)	48.80 (35.70, 65.00)	52.00 (34.45, 68.35)	53.80 (39.70, 69.60)	29.30 (18.30, 42.15)	30.00 (19.95, 43.60)	29.75 (19.60, 42.80)
FAI	2.66 (1.75, 4.01)	2.69 (1.94, 4.19)	2.80 (1.51, 3.58)	8.21 (5.16, 11.70)	7.97 (4.97, 11.45)	8.02 (5.05, 13.95)
LH (IU/L)	5.15 (3.50, 7.90)	5.40 (4.00, 8.40)	5.60 (3.90, 8.10)	11.40 (7.20, 16.35)	12.30 (7.90, 17.10)	12.05 (8.70, 19.10)^c^
FSH (IU/L)	6.65 ± 3.01	6.47 ± 2.27	6.60 ± 2.38	5.96 ± 1.73	5.93 ± 1.91	6.50 ± 3.54^c,d^
LH/FSH	0.82 (0.58, 1.27)	0.93 (0.66, 1.43)^a^	0.94 (0.65, 1.30)	1.98 (1.29, 2.70)	2.04 (1.45, 3.00)	2.17 (1.36, 2.97)^c^
**Metabolic profile^e^**						
Fasting Ins (pmol/L)	53.48 (40.98, 72.30)	58.03 (39.14, 79.52)	54.03 (41.53, 78.31)	90.98 (58.34, 134.35)	83.34 (55.70, 122.02)	86.53 (59.38, 133.00)
2 h-Ins (pmol/L)	281.97 (202.69, 410.07)	304.89 (181.06, 512.12)	292.38 (171.13, 380.87)	594.60 (357.39, 1113.70)	554.84 (322.60, 958.20)	476.15 (321.48, 1020.64)
Fasting Glu (mmol/L)	5.19 ± 0.53	5.25 ± 0.44	5.25 ± 0.39	5.33 ± 0.77	5.33 ± 0.81	5.43 ± 0.93
2 h-Glu (mmol/L)	5.99 ± 1.22	5.95 ± 1.32	5.91 ± 1.26	7.35 ± 2.52	6.86 ± 2.04^c^	7.09 ± 2.68
HOMA-IR	1.83 (1.40, 2.50)	1.93 (1.36, 2.85)	1.88 (1.46, 2.76)	3.09 (1.94, 4.74)	2.82 (1.89, 4.23)	2.88 (2.00, 4.72)
TG (mmol/L)	0.81 (0.65, 1.08)	0.91 (0.72, 1.21)^a^	0.92 (0.61, 1.37)	1.10 (0.82, 1.76)	1.11 (0.79, 1.67)	0.99 (0.74, 1.44)
TC (mmol/L)	4.22 ± 0.81	4.23 ± 0.63	4.37 ± 0.72	4.37 ± 0.77	4.41 ± 0.84	4.59 ± 0.77^c^
HDL-C (mmol/L)	1.52 ± 0.32	1.51 ± 0.33	1.50 ± 0.32	1.38 ± 0.36	1.35 ± 0.33	1.46 ± 0.33^c,d^
LDL-C (mmol/L)	2.34 ± 0.67	2.32 ± 0.57	2.48 ± 0.66	2.50 ± 0.73	2.58 ± 0.79	2.68 ± 0.69^c^
ApoA1 (g/L)	1.46 ± 0.22	1.48 ± 0.21	1.42 ± 0.19	1.42 ± 0.21	1.43 ± 0.22	1.44 ± 0.20
ApoB (g/L)	0.75 ± 0.19	0.74 ± 0.15	0.79 ± 0.17	0.81 ± 0.19	0.83 ± 0.21^c^	0.85 ± 0.20
ApoB/apoA1	0.52 ± 0.15	0.51 ± 0.13	0.57 ± 0.15^a,b^	0.58 ± 0.17	0.60 ± 0.19^c^	0.60 ± 0.17
**Oxidative stress parameters^e^**						
TOS (nmol H_2_O_2_ Equiv./mL)	9.45 (7.47, 12.84)	10.22 (7.76, 14.30)	11.09 (8.63, 14.65)	12.29 (9.07, 16.77)	11.64 (9.02, 16.66)	11.59 (8.50, 17.72)
T-AOC (U/mL/min)	14.42 ± 2.51	14.49 ± 2.88	14.88 ± 2.33	15.63 ± 3.12	15.92 ± 3.06	15.85 ± 2.84
OSI	0.66 (0.49, 0.86)	0.72 (0.54, 1.02)^a^	0.81 (0.50, 1.03)	0.77 (0.57, 1.03)	0.74 (0.60, 1.02)	0.78 (0.51, 1.11)
MDA (nmol/mL)	3.63 ± 1.13	3.77± 1.01	3.68 ± 1.25	4.35 ± 1.26	4.33 ± 1.31	4.43 ± 1.35
GSH (nmol/mL)	1.12 ± 0.24	1.10 ± 0.26	1.14 ± 0.24	1.20 ± 0.26	1.17 ± 0.24	1.18 ± 0.24
TOS/GSH	8.74 (6.59, 11.04)	9.33 (7.14, 12.99)	9.27 (7.13, 12.35)	10.26 (7.38, 13.72)	10.40 (7.52, 14.29)	9.80 (6.83, 14.73)

Values are presented as mean ± s.d. or median (25th–75th percentile value). Comparisons of all parameters between two subgroups were corrected for differences in age and BMI except the parameters of age and BMI.

^a^*P* < 0.05, compared with the AA genotype subgroup in the control group; ^b^*P* < 0.05, compared with the AG genotype subgroup in the control group.^ c^*P* < 0.05, compared with the AA genotype subgroup in the PCOS group;^d^*P* < 0.05, compared with the AG genotype subgroup in the PCOS group; ^e^Controls (AA = 221, AG = 231, GG = 64); PCOS (AA =281, AG = 294, GG = 86).

apoA1, apolipoprotein A1; apoB, apolipoprotein B; BMI, body mass index; DBP, diastolic blood pressure; E_2_, estradiol; FAI, free androgen index; F-G, Ferriman–Gallwey; FSH, follicle-stimulating hormone; Glu, glucose; GSH, glutathione; HDL-C, high-density lipoprotein cholesterol; HOMA-IR, index homeostatic model assessment of insulin resistance; Ins, insulin; LDL-C, low-density lipoprotein cholesterol; LH, luteinizing hormone; MDA, malondialdehyde; OSI, oxidative stress index; SBP, systolic blood pressure; SHBG, sex hormone-binding globulin; T-AOC, total antioxidant capacity; TC, total cholesterol; TG, triglycerides; TOS, total oxidant status; TT, total testosterone; WC, waist circumference.

## Discussion

To the best of our knowledge, this is the first study to explore the relationship between *HMOX2* A-42G and G554A SNPs and PCOS. Despite the absence of any association between the G554A and A-42G genetic variations and the risk of PCOS, we found that these two SNPs were significantly associated with E_2_, LH, FSH, LH/FSH ratio, HDL-C, LDL-C, TC, 2 h-Glu, and apoB levels and the apoB/apoA1 ratio in patients with PCOS. In addition to reproductive hormones and metabolic indicators, the A-42G and G554A genetic variants also had a significant effect on the WC, F-G score, and OSI in the control group. Our findings indicate that *HMOX2* G554A and A-42G variations may be linked to endocrine dysfunction of the reproductive system, metabolic disorders of glycolipids, oxidative stress, and body hair growth. Furthermore, we confirmed that the A-42G SNP of *HMOX2* exists in a relatively high linkage disequilibrium with the G554A SNP.

Hereditary factors, oxidative stress, and metabolic disorders play pivotal roles in the pathogenesis of PCOS (4, 5, 6, 7, 23, 24). Despite significant efforts in recent times to ascertain the genes responsible for susceptibility to PCOS and the identification of numerous genes (6, 34, 35, 36, 37), our understanding of PCOS remains incomplete. HMOX2, which is primarily involved in the homeostasis and catabolism of heme, has metabolism regulatory, antioxidant, anti-inflammatory, and antiapoptotic functions (10, 11, 13, 15). Investigating the genetic variants of *HMOX2* in PCOS could assist in identifying the high-risk genes of the disease and clarifying its etiology and pathogenesis.

*HMOX2* is highly expressed in endothelial cells, brain, and testes (10, 15). HMOX2 is critical for maintaining the dynamic balance of heme and its metabolites as well as the chemosensitivity of carotid bodies to oxygen saturation (11, 15, 16). It has been proposed that HMOX2 mainly controls the homeostasis and bioavailability of heme by acting as a heme buffering factor under physiological conditions, and together with inducible HMOX1, it protects against heme toxicity via enzymatic degradation in a state of excessive heme (11). The protein levels and functions of HMOX2 are regulated by heme levels (38, 39). *Hmox2*-null mice developed insulin resistance, elevated blood pressure, promoted subcutaneous and visceral fat tissue deposition and superoxide production, increased levels of pro-inflammatory cytokines, and decreased serum adiponectin levels (40, 41). *Hmox2* deletion in mice also increased proton leakage and glycolysis in gonadal fat pads and decreased body weight in females but not in males under basal conditions (13). Hemin, an HMOX inducer, attenuated palmitate-induced insulin resistance and TG accumulation in mouse primary hepatocytes and improved hypertriglyceridemia, hyperglycemia, liver steatosis, and insulin resistance in mice fed a high-fat diet (42). In bone marrow macrophages stimulated with IL4 and LPS, the expression of HMOX2 in M2 macrophages was significantly higher than that in M1 macrophages (43). Increased expression of Hmox2 is associated with decreased levels of pro-inflammatory cytokines, TC, and TG as well as atherosclerotic lesions in ApoE^-/-^ mice (43). These results indicate that HMOX2 plays a crucial role in preserving the metabolism and equilibrium of heme and exerts significant effects on glycolipid metabolism, oxidative stress, and inflammation.

*HMOX2* genetic variants may affect the expression and enzymatic activity of HMOX2 (16, 17, 44), thus contributing to the incidence and development of disease. *HMOX2* G554A (rs1051308) SNP is situated in a 3’-untranslated region and may affect the process of translation and the stability of the transcript (17, 45). The G allele of the G554A SNP has been associated with a reduced risk of essential tremors in Spanish population (18). However, it was associated with an increased risk of Parkinson’s disease in Han Chinese men (22). Additionally, the A allele of this SNP is a risk factor for multiple sclerosis in Spanish Caucasian men (19). *HMOX2* A-42G (rs2270363) SNP is located in the 5′ flanking region of *HMOX2* or the intron 1 of the longest transcript of HMOX2 multiple isoforms and may affect the transcription of the target gene by disrupting the binding of transcription factors (20, 44). The GG genotype and G allele of the A-42G SNP were associated with a higher risk of developing Parkinson’s disease in the Spanish population (17). Nevertheless, the A allele of this SNP has been associated with an increased risk of dry age-related macular degeneration in the Polish population (21) and schizophrenia in the Chinese population (20, 44). The present study demonstrated that* HMOX2* A-42G and G554A genetic variants were not correlated with the risk of PCOS in Chinese females.

It has been reported that circulating HMOX1 but not HMOX2 levels are lower in nonobese women with PCOS than those in the healthy control women. HMOX1 levels are negatively associated with TT, LH, insulin resistance, oxidative stress, and inflammation; furthermore, low serum HMOX1 is an independent risk factor for PCOS (46). However, in another study, *HMOX1* mRNA expression in subcutaneous adipose tissue is higher in women with PCOS than in the controls matched for age and BMI. Increased *HMOX1* mRNA levels correlate with a high BMI and insulin resistance, and may be a compensatory mechanism to reduce oxidative stress and inflammatory status in adipose tissue (47). In letrozole-induced PCOS rats, the administration of hemin ameliorated oxidative stress, reduced inflammatory responses, restored hormonal balance, normalized metabolic function, and improved ovarian function (48). Our study found that patients with PCOS with the AA or AG genotype of A-42G SNP had significantly higher E_2_, LH, FSH, LH/FSH ratio, TC, HDL-C, LDL-C, apoB, and/or apoB/apoA1 ratio than those with the GG genotype. Patients with the GG or AG genotype of G554A SNP had higher LH, FSH, E_2_, LH/FSH ratio, HDL-C, LDL-C, TC, apoB, or apoB/apoA1 ratio but lower 2 h-Glu levels in comparison with those carrying the AA genotype. Our findings suggest that the A-42G and G554A genetic variants could play a role in endocrine irregularities in the reproductive system and disturbances in glycolipid metabolism in PCOS. Additionally, we found that the control group of participants with the AA or AG genotype of A-42G SNP had higher apoB/apoA1 ratio, TG levels, or OSI but lower FAI compared with those with the GG genotype. The control participants with the GG or AG genotype of G554A SNP had higher LH/FSH ratio, apoB/apoA1 ratio, TG, or OSI but lower WC compared with those with the AA genotype. Therefore, apart from metabolism and endocrinology, these two SNPs also have an obvious effect on oxidative stress in controls. Our findings suggest that a relatively high linkage disequilibrium between A-42G and G554A SNPs can partially explain the effects of these two SNPs on hormones, glycolipid metabolism, and oxidative stress; however, further research is needed on the mechanisms underlying the effects of these two variants.

The present study has certain limitations. First, owing to insufficient sampling, we were unable to determine HMOX2 activity, which could have provided further evidence to clarify the mechanisms underlying the effects of *HMOX2* A-42G and G554A variants on hormones, metabolism, and oxidative stress. Second, some participants were not evaluated for oxidative stress and hormonal or metabolic indices because confounding factors, which could have affected the statistical effectiveness of these indicators.

In conclusion, although we did not observe any association of *HMOX2* A-42G and G554A genetic variants with the risk of PCOS in a Chinese population, we found that these polymorphisms are linked to endocrine abnormalities of the reproductive axis and glycolipid metabolic disorders in patients. Our findings suggest that *HMOX2* A-42G and G554A polymorphisms may be involved in the pathogenesis of PCOS.

## Declaration of interest

The authors declare no competing interests.

## Funding

This work was funded by the National Natural Science Foundation of Chinahttp://dx.doi.org/10.13039/501100001809 (81370681) and the Program for Chang Jiang Scholars and Innovative Research Team in the University, Ministry of Educationhttp://dx.doi.org/10.13039/501100002701 (IRT0935)

## Author contribution statement

PF, XZ, and SL conceived and designed the experiments; XZ drafted the manuscript; XZ, QL, and CY conducted the experiments; PF and XZ analyzed the data; HL and HB participated in the interpretation or acquisition of data; HL recruited and screened the patients; PF, HB, and SL edited the manuscript; and QL and CY helped with the research. The final draft of the manuscript has been read and approved by all the authors.
